# Transit Peptides Often Require Downstream Unstructured Sequence for Efficient Chloroplast Import in *Chlamydomonas reinhardtii*

**DOI:** 10.3389/fpls.2022.825797

**Published:** 2022-05-12

**Authors:** Oliver D. Caspari

**Affiliations:** Chloroplast Biology and Light Sensing in Microalgae Research Group (UMR7141), Institut de Biologie Physico-Chimique, CNRS/Sorbonne Université, Paris, France

**Keywords:** disordered protein, protein import, chloroplast, targeting peptide, transit peptide, presequence, *Chlamydomonas reinhardtii*

## Abstract

The N-terminal sequence stretch that defines subcellular targeting for most nuclear encoded chloroplast proteins is usually considered identical to the sequence that is cleaved upon import. Yet here this study shows that for eight out of ten tested Chlamydomonas chloroplast transit peptides, significant additional sequence stretches past the cleavage site are required to enable efficient chloroplast import of heterologous cargo proteins. Analysis of Chlamydomonas cTPs with known cleavage sites and replacements of native post-cleavage residues with alternative sequences points to a role for unstructured sequence at mature protein N-termini.

## Introduction

Chloroplasts derive from once free-living cyanobacteria and retain genomes of their own, yet the vast majority of genes coding for plastid proteins is located in the nucleus (Shi and Theg, 2013). Proteins are expressed in the cytosol as preproteins containing N-terminal chloroplast targeting peptides (cTP), which direct import into the chloroplast (Chotewutmontri et al., 2017). Upon import, the N-terminus of the preprotein is cut off at a defined cleavage site and degraded (Teixeira and Glaser, 2013; Kmiec et al., 2014).

The cTP, i.e., the sequence both necessary and sufficient to generate chloroplast targeting, is generally assumed to coincide with the sequence stretch that is cleaved off. It is this cleaved presequence that has been studied for decades, with a well-documented tripartite structure and multiple sequence elements now recognized to contribute to targeting (von Heijne et al., 1989; Chotewutmontri et al., 2017; Caspari and Lafontaine, 2021). There is very little sequence conservation between cTPs, instead targeting information is encoded through particular physico-chemical properties exclusive to cTPs (Bruce, 2001). The N-terminus of cTPs is thought to be of particular importance in preventing mistargeting to the mitochondria (Bhushan et al., 2006). Relatively uncharged in plant cTPs (Chotewutmontri et al., 2012), the presence of multiple arginines in mTP N-termini was found to prevent chloroplast import (Lee et al., 2019). In Chlamydomonas, cTP N-termini are less uncharged and more unstructured, but retain key physico-chemical attributes (Caspari et al., 2021). Hsp70-binding sites where shown to be present in cTP N-termini and be important for import (Chotewutmontri and Bruce, 2015). The central part of cTPs is overall positively charged due to the presence of arginines (Caspari et al., 2021). While generally unstructured (von Heijne and Nishikawa, 1991), this part carries amphipathic helix signatures (Garrido et al., 2020) and may fold into such helices upon contact with a membrane (Bruce, 2000). Indeed, membrane interactions of cTPs can be crucial for targeting (Pinnaduwage and Bruce, 1996). At the C-terminus, cTPs carry recognition sites for cleavage by the stromal processing peptidase upon completion of chloroplast import, the only part of cTPs that shows weak sequence conservation (Tardif et al., 2012; Teixeira and Glaser, 2013). Across the length of cTPs, various protein interaction motifs can be found. Rich in serines, phosphorylation of cTPs is thought to play a role in regulation of import and in enabling interaction with 14-3-3 (Sjuts et al., 2017), a chaperone that forms a cytosolic guidance complex with Hsp70 (May and Soll, 2000). Along with Hsp90, such cytosolic chaperones can help to prevent the folding of cargo proteins, which is particularly important for hydrophobic membrane proteins, and interact *via* receptors such as the tetratricopeptide Toc64 to deliver preproteins to the chloroplast import machinery (Chotewutmontri et al., 2017; Sjuts et al., 2017). A different set of receptors, notably Toc159 and Toc34, also bind cTPs directly. Semi-conserved FGLK-motifs have been suggested as cis-elements that may mediate TOC-interactions (Chotewutmontri et al., 2017), although the F may be less strictly required in Chlamydomonas (Razzak et al., 2017; Caspari et al., 2021). Thus, plenty of evidence exists that chloroplast targeting, in the sense of differential protein sorting, is encoded within the cleavable cTP.

Yet sequence stretches that lie beyond the cleavage site may also contribute to targeting, especially in the sense of import efficacy. Parts of the mature protein N-terminus have been recognized before as a requirement for efficient targeting in vascular land plants. A number of studies have noted a requirement for extending different cTPs into the mature sequence to achieve efficient import of heterologous cargo proteins (Comai et al., 1988; Rolland et al., 2016; Shen et al., 2017). Bionda et al. (2010) described a length requirement of ~55–60 amino acids for *Arabidopsis thaliana* and proposed that in the ~50% of chloroplast targeted proteins where the cleavable part of the cTP is shorter, residues C-terminal of the cleavage site contribute unstructured sequence toward targeting. There are even examples of import through the canonical TOC/TIC system of proteins lacking a cleavable N-terminal presequence altogether (Chang et al., 2014), implying the entire targeting information is present in the mature protein.

It was noted decades ago that cleavable cTPs are generally shorter in the model green alga *Chlamydomonas reinhardtii* compared to vascular land plants based on the placement of cleavage sites (Franzén et al., 1990), suggesting there may be a role for mature protein N-termini. In Chlamydomonas, the Rubisco activase (RCA1) cTP has recently been found to require post-cleavage site residues to target a fluorescent reporter protein to the chloroplast (Garrido et al., 2020). Targeting of a fluorescent reporter had previously been achieved using the Rubisco small subunit (RBCS) 2 cTP from Chlamydomonas extended by 23 residues (Razzak et al., 2017). Plant RBCS-cTPs were equally found to require extension by 24 residues into the mature protein sequence to enable chloroplast targeting of a heterologous cargo protein (Comai et al., 1988). This present study revisits the role of post-cleavage site residues and finds that stretches of unstructured sequence appear to play a role in enabling efficient chloroplast import by many Chlamydomonas cTPs.

## Materials and Methods

Constructs were Gibson assembled as described in Garrido et al. (2020) based on pMO611, a variant of published pMO449 (Onishi and Pringle, 2016) where an upstream start codon was mutated to CTG. Sequences inserted upstream of Venus were amplified from Chlamydomonas genomic DNA extracted from wild-type strain T222+, with the following exceptions: construct “RBCS2-cTP + 23” was amplified from cDNA generated from T222+ to avoid introducing an intron not present in the other RBCS2-cTP constructs; the G-rich artificial linker sequence was amplified from plasmid CrRaf1-pJHL encoding Raf1-strep (Wietrzynski et al., 2021). Corresponding amino acid sequences are given in Table 1. Constructs were verified by sequencing (Eurofins). Transformants were obtained and microscopy performed as described before (Garrido et al., 2020). For quantification of epifluorescence images, ten cells per construct and their chloroplasts (based on chlorophyll autofluorescence) were outlined manually in Fiji version 1.53q (Schindelin et al., 2012), areas and mean Venus channel intensities recorded, and cytoplasmic intensities inferred. Immunoblotting was performed as described elsewhere (Caspari et al., 2021). For disorder analysis, Chlamydomonas cTPs with characterized cleavage sites were obtained from the literature (Terashima et al., 2011; Tardif et al., 2012) and IUPRED3 (short) disorder scores obtained using the webserver (Erdos et al., 2021). Sequences are listed in Supplementary Table 1. Epifluorescence quantification and sequence disorder results were plotted with R version 3.6.1 (R Core Team, 2019) using R studio version 1.2.1335 (RStudio Team, 2020). Final figures were assembled in PowerPoint (Microsoft Powerpoint for Mac 2011, version 14.7.7). To test for statistical significance, student *t*-tests were used whenever two samples were compared; paired *t*-tests were used where samples are linked; ANOVA followed by Tukey’s honestly significant difference *post-hoc* test was used whenever more than two samples were compared. Tests were done in R.

**Table 1:** Amino acid sequences.

Element	Amino acid sequence
RBCS-cTP (+23)	MAAVIAKSSVSAAVARPARSSVRPMAALKPAVKAAPVAAPAQANQ.MMVWTPVNNKMFETFSYLPPLSD
RBCA-cTP (+23)	MQVTMKSSAVSGQRVGGARVATRSVRRAQLQVV.ASSRKQMGRWRSIDAGVDASDDQ
CAH4-mTP	MSSRNVATALRMFATLGRSQAGEASAMMGTGSALLAQRAAALGGASAVNKGCSCRCGRVACMGACMPMRH.
CAG2-mTP	MLKRVGQSLVPFARAGLTQTAESFRG.
γATPs-cTP (+5)	MAAMLASKQGAFMGRSSFAPAPKGVASRGSLQVVA.GLKEV
CP29-cTP (+5)	MVFKFPTPPGTQKKAGTTATKPAPKATTKKVATSTGTRSGGVGYRKY.QGDAL
FD-cTP (+5)	MAMAMRSTFAARVGAKPAVRGARPASRMSCMA.YKVTL
LHCBM4-cTP (+5)	MAFALATSRKALQVTC.KATGK
PGP1-cTP (+5)	MVAAQASARPIATNEQKLELLKKVECFI.FDCDG
PRPL4-cTP (+5)	MQTMRVAFRPAATSRSTVVTRA.SAVAA
PSRP3-cTP (+5)	MMLVRQVPCLAQRASARSAVRSRPAPFTPARRVFARAQADAALLEDDEVAYEVTNEDFELDENDPDLLLGLEAVELMGSDVDAM.TAAAV
PSAD-cTP (+5)	MAVMMRTQAPAATRASSRVAVAARPAARRAVVVRA.EAEAA
+EPYC1	SSSPAPASSAPAPARSSSA
+VIPP1	SAVGSLPEPRAVDALDLEL
+linker	SGGGGSGGGGSMGGGGSN
+RBCXa	KTMEMASRETREANTRLM

Cleavage sites are indicated with a full stop between residues

## Results

**Figure 1:**
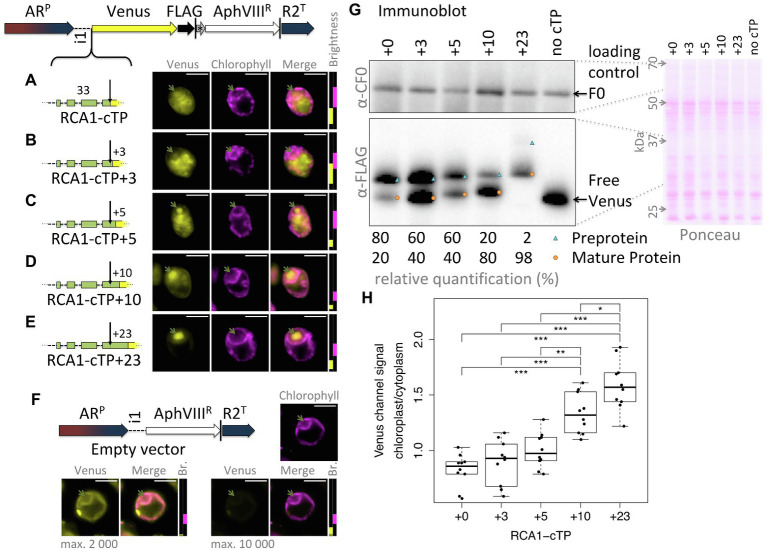
RCA1-cTP relies on residues past the cleavage site to support efficient chloroplast import. **(A–E)** Constructs were generated by inserting candidate peptides upstream of Venus in a bicistronic expression vector made of the following components: AR^P^: Hybrid *HSP70A*-*RBCS2* promoter, i1: *RBCS2* intron 1, Venus: Venus fluorescent protein (a YFP variant), FLAG: triple-FLAG tag, |: stop codon, ^*^: bicistronic intergenic sequence (tagcat), AphVIII^R^: Paromomycin resistance gene, and R2^T^: *RBCS2* terminator. Here, candidate peptides are the Rubisco activase (RCA1) chloroplast targeting peptide (cTP), extended by **(A)** +0, **(B)** +3, **(C)** +5, **(D)** +10, or **(E)** +23 residues beyond the cleavage site into the mature protein. Lengths in amino acid residues are indicated above peptide cartoons. Introns within peptides are symbolized by dashed lines. Typical epifluorescence images of resulting transformants in strain T222^+^ show fluorescence from the Venus channel in yellow and chlorophyll autofluorescence in magenta. The position of the pyrenoid is highlighted by a green arrow in each image. All scale bars are 5 μm in length. Image brightness (Br.) was adjusted for clarity by restricting the visualized intensities to a subset of recorded intensity values, as shown to the right of micrographs (intensity 0 at bottom, 65535 at the top; 2000 indicated by a black dotted line). **(F)** A control transformed with an empty vector shows the extent of autofluorescence in the Venus channel. **(G)** Whole-cell protein extracts were normalized to 5 μg chlorophyll per lane. Ponceau-red staining of the membrane used for blotting gives an indication of equal loading and the quality of the transfer. The mitochondrial ATPsynthase subunit F0, immunolabeled with an antibody raised against chloroplast ATPsynthase subunit CF0, is shown as a loading control. FLAG was immunolabeled to visualize the migration of the Venus reporter in each strain. Preprotein (labeled with a blue triangle) migrates above mature protein that had the cTP cleaved upon chloroplast import (labeled with an orange circle). A relative quantification was done on a less exposed image; a slightly overexposed image was chosen as visual representation to show the presence of preprotein in the RCA1-cTP + 23 strain. **(H)** Epifluorescence images were quantified by measuring mean Venus channel signal inside and outside the chloroplast. Statistical significance is indicated as: ^*^p < 0.05, ^**^p < 0.01, and ^***^p < 0.001;Tukey’s honestly significant difference *post-hoc* test, after ANOVA.

This study was seeded by the observation that the full-length RCA1-cTP fails to generate reliable chloroplast localization of a Venus fluorescent reporter, expressed from a bicistronic expression vector (Onishi and Pringle, 2016; Caspari, 2020), in Chlamydomonas (Figure 1A): fluorescence signal in the Venus channel was visible from across the cell, rather than being restricted to the chloroplast, the compartment labeled by chlorophyll autofluorescence. Given that successful targeting using the RBCS2-cTP had previously been reported when extended by 23 residues downstream of the cleavage site in Chlamydomonas (Razzak et al., 2017) and by 24 in vascular land plants (Comai et al., 1988), and extending the RCA1-cTP by 23 residues had equally restored targeting (Garrido et al., 2020), a series of successive extensions was prepared. RCA1-cTP constructs extended by +3 and + 5 residues both show significant Venus fluorescence from outside the chloroplast (Figures 1B,C). The RCA1-cTP + 10 construct shows reduced Venus signal in the cytoplasm (Figure 1D). Finally, the +23 construct shows clear Venus signal inside the chloroplast only (Figure 1E).

When judging Venus localization in the chloroplast based on fluorescence micrographs, it should be noted that a small amount of autofluorescence is visible in the Venus channel even in cells that do not express the Venus reporter (Figure 1F). While brightest in the eyespot, most of this autofluorescence colocalizes with chlorophyll fluorescence and may thus be confused with chloroplast localization of the reporter. Two indicators are used to identify true Venus localization to the chloroplast: Firstly, autofluorescence is low (usually <2000 intensity units). Whenever possible, the brightness of the images has therefore been adjusted to screen out autofluorescence; for transpareny, brightness settings for each image are shown next to the merged image with a dotted black line showing the 2000 intensity units mark (Figure 1F). Secondly, when Venus accumulates in the chloroplast, Venus fluorescence is visible from inside the pyrenoid, a proteinaceous structure that generates a visible dip in chlorophyll fluorescence (Mackinder et al., 2017). Venus channel autofluorescence equally dips in the pyrenoid (Figure 1F). Note that the RCA1-cTP + 23 construct contains a pyrenoid-localization motif (Meyer et al., 2020), making pyrenoid accumulation particularly pronounced in this construct (Figure 1E). However, even without this motif, Venus fluorescence from within the chloroplast appears brightest in the pyrenoid region (Figures 1A–D). It is unclear why Venus should accumulate in the pyrenoid, beyond the fact that as a relatively small protein it is not excluded from the pyrenoid matrix (Freeman Rosenzweig et al., 2017). Any apparent attraction toward the pyrenoid does not appear to be very strong, as the same Venus reporter sequence has been used to localize different proteins to a number of chloroplast subcompartments (Mackinder et al., 2017). It is possible that the signal is simply strongest from within the pyrenoid due to absorption of part of the fluorescence by chlorophyll in other parts of the chloroplast. However, an in-depth investigation of this phenomenon was beyond the remit of this study; here, Venus signal from within the pyrenoid was simply used as a visual guide to true chloroplast localization of the reporter.

An immunoblot (Figure 1G) was used to ascertain whether the partial chloroplast targeting seen *via* microscopy correlated with partial maturation of the preprotein. A loading control and a Ponceau-red stain attest to relatively equal loading of the samples. The Venus reporter was immunolabeled with an antibody against FLAG. RCA1-cTP constructs each show two bands that migrate at increasing apparent molecular weight, matching the sequence extensions. In each case, the upper band corresponds in size to the preprotein and the lower band to the mature protein after maturation at the annotated cleavage site. A control expressing the reporter with no cTP shows where free Venus migrates. The presence of a mature protein fraction in the unextended RCA1-cTP construct, migrating at the same size as free Venus, indicates partial targeting of the reporter to the chloroplast and is in line with part of the Venus fluorescence signal emanating from within the pyrenoid in this construct (Figure 1A). Comparing the immunoblot signal intensity provides a rough quantification of the fraction of preprotein that has been cleaved (Figure 1G): while without extension, this corresponds to a minority of 20% in the RCA1-cTP construct, the fraction increases to 40% in the +3 and + 5 constructs, to 80% in the +10 construct, and to 98% in the +23 construct.

Quantification of the epifluorescence signal in the chloroplast relative to the cytoplasm across ten cells per strain (Figure 1H) shows a similar trend. The unextended RCA1-cTP construct (+0) shows values below 1, signifying that cytoplasmic Venus outweighs both Venus and autofluorescence emanating from the chloroplast. Values increase as the cTP is extended beyond the cleavage site, with the +10 construct showing significantly higher values and the +23 construct being higher still.

**Figure 2:**
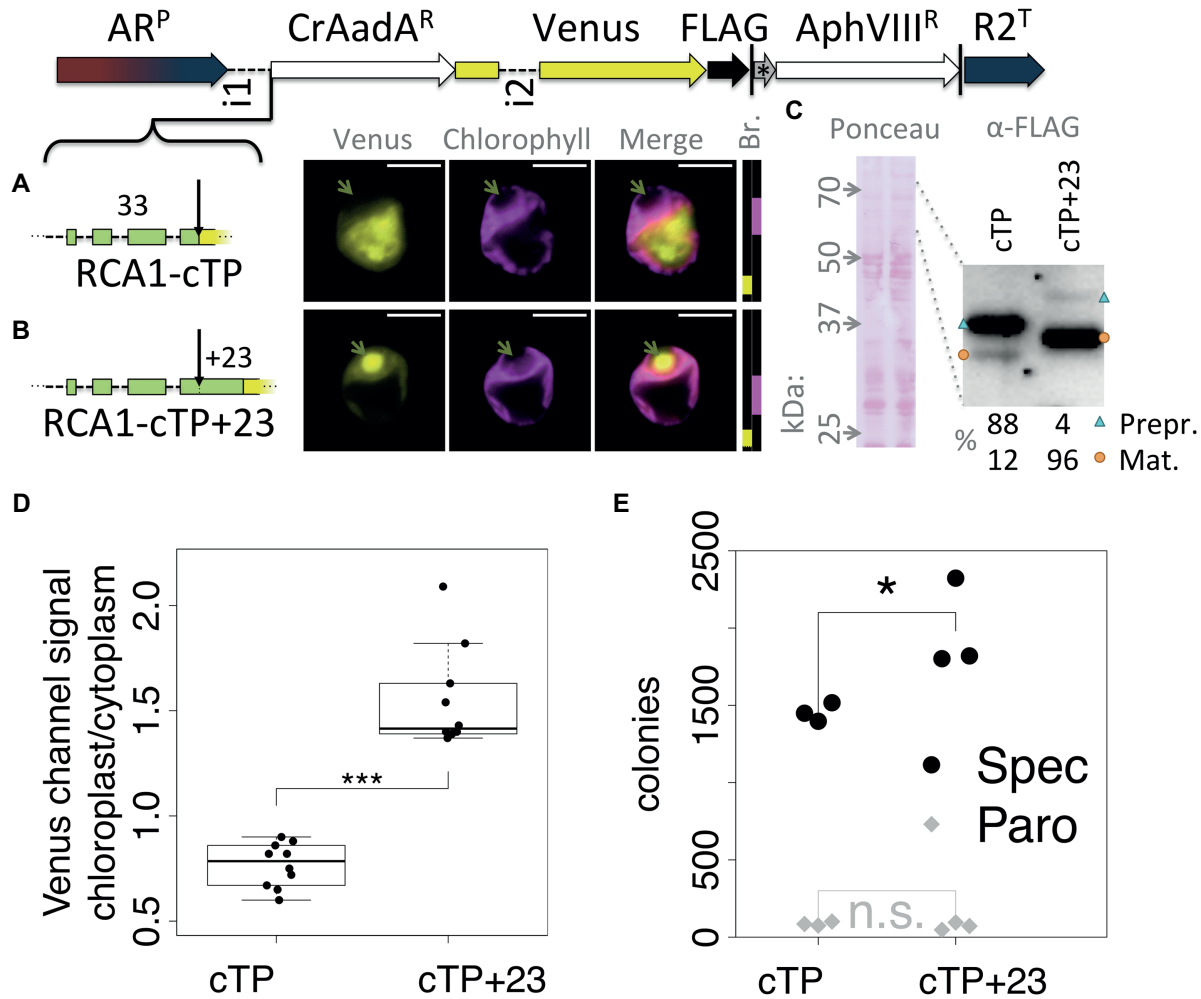
An alternative cargo confirms the requirement for extending RCA1-cTP past the cleavage site. **(A–C)** The spectinomyin resistance gene AadA, codon optimized for Chlamydomonas (CrAadA^R^) and equipped with a short linker sequence at the C-terminus, was inserted between RCA1-cTP ± 23 and Venus, and transformants analyzed using fluorescence microscopy and immunoblotting. Green arrows highlight the pyrenoid in **(A,B)**; blue triangles highlight preprotein bands; orange circles highlight mature protein bands in **(C)**; and see Figure 1 caption for further details on representation. **(D)** Epifluorescence images were quantified by measuring mean Venus channel signal inside and outside the chloroplast (^***^signifies *p <* 0.001; *t*-test). **(E)** Wild-type (T222+) cultures were transformed with either construct in triplicate, and each transformation mix split such that part was plated on 20 μg ml^−1^ Paromomycin plates and part on 1 mg ml^−1^ Spectinomycin plates. The plot shows the number of colonies obtained (^*^signifies *p* < 0.05; Tukey’s honestly significant difference *post-hoc* test, after ANOVA).

To ensure that the observed effect was not particular to the Venus reporter, a version of the Spectinomycin resistance gene AadA that was codon optimized for Chlamydomonas (Meslet-Cladière and Vallon, 2011) was inserted between RCA1-cTP and Venus (Figure 2). In this construct, RCA1-cTP resulted in strong signal from outside the chloroplast (Figure 2A), whereas RCA1-cTP + 23 gave rise to clear chloroplast localization with a strong signal from within the pyrenoid (Figure 2B). Relative quantification of an immunoblot (Figure 2C) suggests that 88% of preprotein remains uncleaved in the absence of the post-cleavage site extension, whereas 96% are imported and cleaved in the RCA1-cTP + 23 construct. Quantification of epifluorescence images shows a ratio of chloroplastic to cytoplasmic Venus channel signal below 1 for the RCA1-cTP construct, consistent with a majority of Venus present in the cytoplasm. Significantly higher values above 1 are seen for the RCA1-cTP + 23, consistent with chloroplast localization (Figure 2D). The CrAadA gene was reported to provide spectinomycin resistance even when expressed from the nuclear genome, but this resistance is improved when the protein is imported into the chloroplast (Meslet-Cladière and Vallon, 2011). Consistently, the number of colonies obtained after selection on 1 mg ml^−1^ spectinomycin plates was significantly higher in the RCA1-cTP + 23 construct (Figure 2D, *p =* 0.012, Tukey’s honestly significant difference *post-hoc* test following ANOVA). There was no difference after selection for paromomycin resistance (Figure 2D, *p >* 0.99) among cells from the same electroporation reaction mix, as expected for this control for the absence of other biases in the experiment.

**Figure 3:**
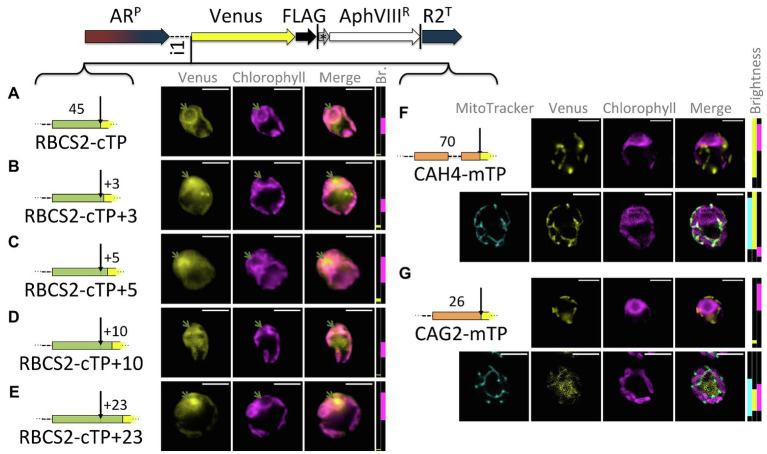
RBCS2-cTP shows a similar reliance on sequence extension, in contrast to mTPs. **(A–E)** Rubisco small subunit 2 (RBCS2) cTP and extensions were used to drive Venus localization as in Figure 1; green arrows highlight pyrenoid location. **(F,G)** mTPs of mitochondrial carbonic anhydrase 4 (CAH4) and γ-carbonic anhydrase 2 (CAG2) were assessed by epifluorescence as before, and also by confocal fluorescence microscopy in the presence of a MitoTracker dye (false-colored in cyan) to provide a visual co-localization guide to assess mitochondrial localization of Venus.

Extensions of the RBCS2-cTP show a similar trend as those of RCA1-cTP, despite lower Venus signal (Figure 3): the sequence up to the cleavage site generates very low Venus fluorescence from within the pyrenoid but does show signal from within the cytoplasm (Figure 3A). Extension by +3 and + 5 residues show both signal from the pyrenoid region and from outside the chloroplast (Figures 3B,C). The RBCS2-cTP + 10 construct has a very low signal, which is present in both cytoplasm and chloroplast (Figure 3D). Only the RBCS2-cTP + 23 construct shows no signal in the cytoplasm while also showing a clear Venus signal from within the pyrenoid, demonstrating efficient chloroplast import of the reporter. Note that the Venus signal was too close to autofluorescence to allow meaningful quantification of the epifluorescence images between these constructs.

By contrast, mitochondrial targeting peptides (mTPs) from carbonic anhydrase 4 (CAH4) and γ-carbonic anhydrase 2 (CAG2) show mitochondrial targeting, evident as co-localization of Venus and MitoTracker fluorescence, in the absence of any post-cleavage site residues (Figures 3F,G). CAG2-mTP shows some remaining signal from the cytoplasm, which persists when the construct is extended by 23 residues past the cleavage site (Garrido et al., 2020).

**Figure 4:**
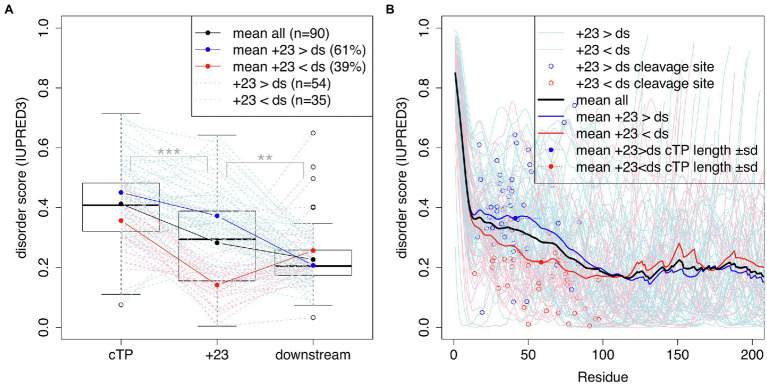
cTPs and post-cleavage site stretches are more disordered than downstream mature protein sequences. Protein disorder profiles of 90 chloroplast-localized, nuclear encoded Chlamydomonas proteins with known cleavage sites were obtained using IUPRED3. Disorder scores can range from 0 to 1 with higher values implying more disorder. **(A)** Boxplots show the distribution of scores for sequences up to the cleavage site (cTP), the 23 residues following the cleavage site (+23) and mature protein sequences excluding the N-terminal 23 residues (downstream; ds). Statistical significance is shown as ^***^ for *p* < 0.001 and ^**^ for *p* < 0.01 (paired *t*-tests). Values for individual sequences are shown in dashed lines, in blue when the +23 value is higher than the downstream value, or in red otherwise (one case where the sequence past the cleavage site was less than 23 residues long is in shown in gray). **(B)** Individual and mean disorder profiles along the sequence are shown starting from preprotein N-termini. The position of the cleavage site is highlighted by showing the last cTP residue disorder value as a point.

Bionda et al. (2010) suggested that mature protein N-termini may contribute unstructured sequence to the targeting of short plant cTPs. To assess whether a similar dynamic may be at play in algal cTPs, protein disorder along the sequence was predicted for a set of 90 Chlamydomonas cTPs with known cleavage sites (Figure 4) using the IUPRED3 (short) algorithm (Erdos et al., 2021). Figure 4A: While significantly less disordered than the cTP up to the cleavage site ( $p=8.1\times 10^{-13}\text{,}$ paired *t*-test), the stretch of 23 residues immediately following the cleavage site (+23) is significantly more disordered than downstream (ds) mature protein sequences ( p=0.0037 , paired *t*-test). There is heterogeneity underneath this overall pattern: While a majority of sequences (61%) individually show higher disorder in the +23 region than downstream, a minority (39%) show the opposite pattern (Figure 4A). Overall, preprotein sequences show a higher degree of disorder within the *ca.* 50 N-terminal residues than in the main part of mature protein sequences past *ca.* 100 residues (Figure 4B). Sequences where the +23 stretch is more disordered than downstream parts of the mature protein (in blue) tend to have shorter cTPs (41.3 ± 17.9 residues) than sequences with less disordered mature protein N-termini (58.5 ± 23.1 residues; *p* = 0.00039, *t*-test). While the latter on average show lower disorder within the cTP, sloping down to the mature protein level upstream of the cleavage site, the former maintain high disorder within the cTP and slope down in the mature protein N-terminus (Figure 4B). Unstructured sequence past the cleavage site may thus indeed play a part in targeting in Chlamydomonas, at least for a subset of cTPs.

**Figure 5:**
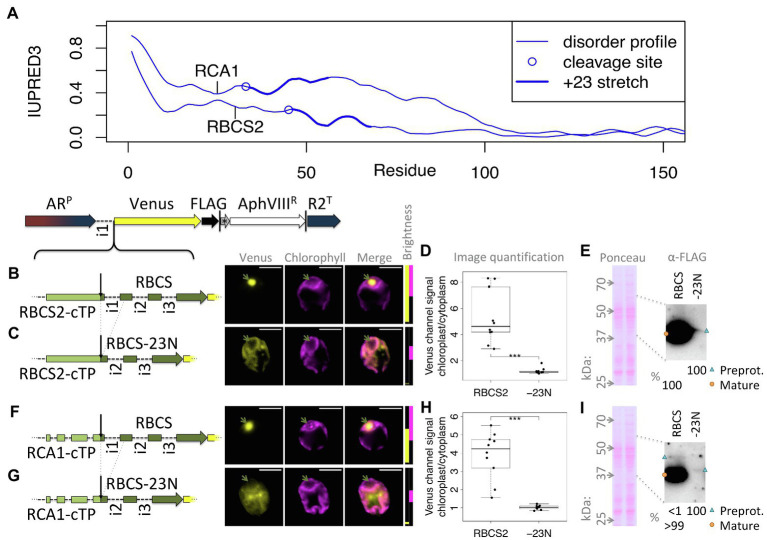
Deleting mature protein N-termini disrupts chloroplast import and reporter accumulation. **(A)** IUPRED3 disorder profiles are shown for RCA1 and RBCS2 preprotein sequences as in Figure 4B, with the +23 stretch highlighted. **(B)** RBCS2, equipped with the native RBCS2-cTP, was introduced upstream of Venus and transformants imaged as in Figure 1. **(C)** A “-23 N” construct was generated and imaged as before where the sequence encoding the N-terminal 23 residues of RBCS2 was deleted, which includes intron 1. **(D)** Epifluorescence images were quantified by measuring mean Venus channel signal inside and outside the chloroplast (^***^ signifies *p <* 0.001; *t*-test). **(E)** Immunoblot and corresponding Ponceau-red stained membrane are shown comparing full-length RBCS2 and the truncated -23 N construct, both driven by RBCS2-cTP. **(F)** full-length and **(G)** truncated (-23 N) versions of RBCS2 where fused to the RCA1-cTP, and analyzed by microscopy, epifluorescence quantification **(H)**, and **(I)** immunoblot as before. Green arrows highlight the pyrenoid, blue triangles preprotein bands, and orange circles mature protein bands.

One test of the importance of mature protein N-termini is to assess targeting after deletion of the stretch suspected to contribute to targeting. Figure 5A shows that both RCA1 and RBCS2 preproteins show higher disorder in the +23 region than in the downstream sequence. Yet RCA1 maintains increased disorder for another *ca.* 40 residues beyond the +23 stretch, which might compensate for deletion of the N-terminus with respect to enabling chloroplast import. Therefore, fusions of both RBCS2-cTP and RCA1-cTP to RBCS2 were used to assess mature protein N-terminal deletions (Figures 5B–G). An RBCS2-Venus fusion protein, targeted by the native RBCS2-cTP, shows accumulation in the pyrenoid (Figure 5B), matching the native localization of the RBCS2 protein (Mackinder et al., 2016). Upon deletion of 23 residues from the RBCS2 mature protein N-terminus (-23 N), signal is very low but Venus channel fluorescence is absent from the pyrenoid region and present outside the chloroplast (Figure 5C), suggesting the construct fails to support import into the chloroplast. Quantifying the images (Figure 5D) backs up significant Venus accumulation of native RBCS2 in the chloroplast. By contrast, values for the truncated version are close to 1, consistent with low level Venus accumulation in the cytoplasm counterweighing autofluorescence from the chloroplast. Upon long exposure, a small amount of the -23 N construct can be seen migrating slightly above the full-length RBCS band in an immunoblot (Figure 5E), consistent with an efficiency of import/cleavage near 100% in the presence of the +23 stretch and a complete absence of import/cleavage upon deletion. Using the RCA1-cTP to target the RBCS2-Venus fusion protein shows a similar pattern (Figures 5F–I): Clear import is observed with full-length RBCS2 (Figure 5F), cytoplasmic Venus is observed in the -23 N construct (Figure 5G). Image quantification (Figure 5H) supports chloroplast accumulation of full-length RBCS2 and cytoplasmic accumulation of the -23 N construct. Immunoblot data (Figure 5G) support import/cleavage of >99% of full-length RBCS2 while no cleavage can be seen upon deletion of the N-terminus. It is of note that for both cTPs, deletion of the mature RBCS2 N-terminus disrupted not just chloroplast import, but also severely affected protein accumulation. RBCS2 intron 1 is known to promote expression, and a copy of this intron is contained within the deleted N-terminus, which may be expected to lead to lower expression. Misfolding of the truncated RBCS2 may also promote degradation. This low level of reporter accumulation may mask partial chloroplast targeting, if the fraction that successfully imports into the chloroplast is too small to be detected in either the microscopy or the immunoblots.

**Figure 6:**
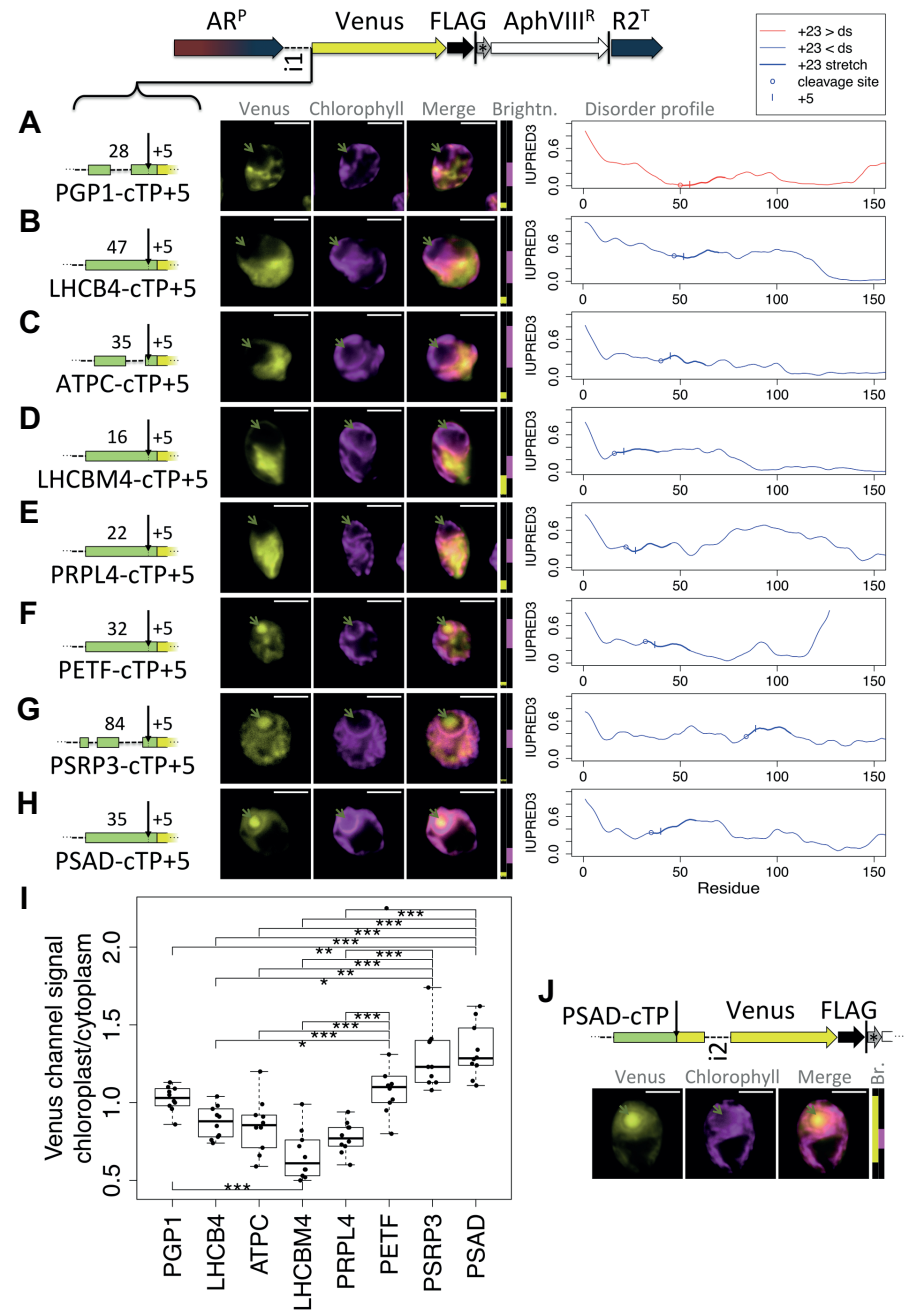
Efficient chloroplast import relies on post-cleavage site sequence in many cTPs. Candidate chloroplast targeting peptides, each including 5 post-cleavage site residues, from **(A)** phosphoglycolate phosphatase (PGP1), **(B)** chlorophyll a/b binding protein of photosystem II, also known as CP29 (LHCB4), **(C)** chloroplast ATP-synthase gamma chain (ATPC), **(D)** chlorophyll a/b binding protein of light harvesting complex II (LHCBM4), **(E)** plastid ribosomal protein L4 (PRPL4), **(F)** chloroplast ferredoxin (PETF), **(G)** plastid-specific ribosomal protein 3 (PSRP3), and **(H)** Photosystem 1 subunit D (PSAD), were inserted upstream of Venus. Individual IUPRED3 disorder profiles are shown as in Figure 4B, with the +23 stretch highlighted and the +5 residue marked by |. **(I)** Epifluorescence images were quantified by measuring mean Venus channel signal inside and outside the chloroplast. Statistical significance is indicated as: ^*^p < 0.05, ^**^p < 0.01, and ^***^p < 0.001; Tukey’s honestly significant difference *post-hoc* test, after ANOVA. **(J)** A second PSAD construct lacking post-cleavage site residues was probed. Lengths in amino acid residues are indicated above each peptide; green arrows show pyrenoid location; see Figure 1 for further details on representation.

As a second test for whether the presence of a relatively long stretch of mature protein is a general requirement, a further eight cTPs were assayed for import (Figure 6). These cTPs were each extended by 5 residues to ensure any lack of import was not an artifact arising from a missing cleavage site, for which several residues past the cleavage site seem to be important (Tardif et al., 2012). The PGP1-cTP + 5 fails to show chloroplast import (Figure 6A). This sequence has lower disorder in the +23 stretch than downstream, suggesting that even among such sequences, the mature protein may play a part in import. The cTP + 5 constructs from LHCB4, ATPC, LHCBM4, and PRPL4 (Figures 6B–E) all show high Venus signal from outside the chloroplast, and no signal from within the pyrenoid, indicating that these sequences may be even more reliant on post-cleavage site residues than RCA1 (Figure 1) or RBCS2 (Figure 3) that both showed partial import with a + 5 extension. A PETF construct (Figure 6F) shows Venus signal from within the pyrenoid as well as from outside the chloroplast, consistent with partial targeting as seen for RCA1 and RBCS2. The PSRP3 and PSAD constructs appear capable of generating fully chloroplast-localized Venus (Figures 6G,H). Quantification of the epifluorescence images (Figure 6I) shows chloroplast/cytoplasm ratios of 1 or less for PGP1, LHCB4, ATPC, LHCBM4, and PRPL4, consistent with cytoplasmic Venus accumulation; PETF shows significantly higher values that are slightly above 1, consistent with partial chloroplast targeting; PSRP3 and PSAD show the highest values, consistent with chloroplast localization of the fluorescent reporter. Chloroplast targeting with the PSAD-cTP also works in the absence of any post-cleavage site residues (Figure 6I). Thus of ten cTP + 5 constructs tested (Figures 1, 3, 6), nine of which were from sequences with higher disorder in the +23 stretch than downstream (Figures 5A, 6B–H), five (RCA1, RBCS, PETF, PSRP3, and PSAD) showed evidence that at least some of the Venus reporter is imported into the chloroplast, and two (PSRP3 and PSAD) showed efficient import with little remaining cytoplasmic signal.

**Figure 7:**
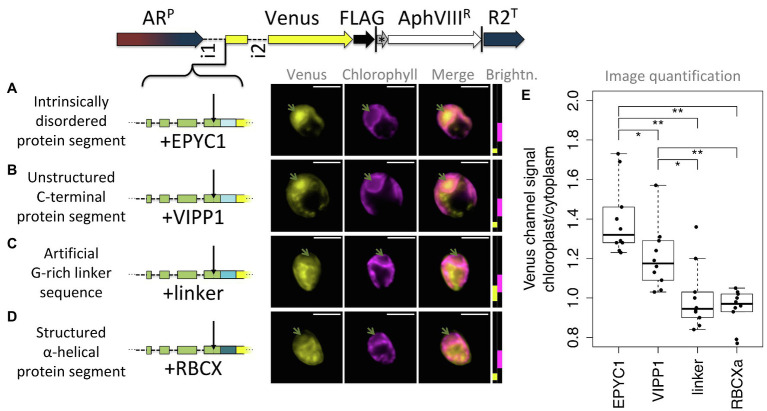
Unstructured downstream sequence aids cTP targeting. The following candidate sequences were inserted downstream of RCA1-cTP + 5 and upstream of Venus: **(A)** residues 195–213 of intrinsically disordered protein EPYC1, which are part of the 3rd out of 4 repeats; **(B)** residues 260–278 of VIPP1, which form part of an unstructured C-terminus; **(C)** an artificial linker sequence mainly consisting of quintuple-G interspersed with S; **(D)** residues 135–154 of RBCXa, which form part of the most C-terminal α-helix. Refer to Table 1 for amino acid sequences. Green arrows show pyrenoid location; see Figure 1 for further details on representation. **(E)** Epifluorescence images were quantified by measuring mean Venus channel signal inside and outside the chloroplast. Statistical significance is indicated as: ^*^p < 0.05; ^**^p < 0.01; Tukey’s honestly significant difference *post-hoc* test, after ANOVA.

As a third test for the hypothesis that post-cleavage site residues contribute unstructured sequence toward chloroplast targeting, four constructs were prepared that each extend the RCA1-cTP + 5 by different sequences (Figure 7). Five mature RCA1 residues were kept to ensure the cleavage site would be maintained intact. Three constructs contained unstructured sequence downstream (Figures 7A–C) and were expected to generate chloroplast targeting, whereas a fourth (Figure 7D) contained an α-helical stretch that was expected to impede chloroplast import. Two of the unstructured sequences as well as the helical stretch were copied from Chlamydomonas nuclear genes encoding chloroplast-localized proteins EPYC1 (also known as LCI5), VIPP1, and RBCXa, respectively. In each of these cases, sequence was chosen from the C-terminal half of the protein, to avoid any sequence that might contribute to the cTPs of these proteins. In addition, a standard G-rich linker sequence, such as is commonly used to join elements when generating fusion proteins in molecular biology, was used to test whether an artificial flexible sequence could substitute for a native unstructured one.

Of these, natively unstructured sequences from the third repeat of intrinsically disordered EPYC1 and the disordered C-terminus of VIPP1 generated chloroplast localization of the Venus reporter (Figures 7A,B). Note that the VIPP1-derived sequence also shows additional Venus signal emanating from cytosolic structures (Figure 7B). By contrast, both the artificial flexible linker and the C-terminal α-helix of RBCXa resulted in only a small amount of Venus localizing to the chloroplast, with a majority of signal emanating from structures outside the chloroplast (Figures 7C,D). Image quantification corroborates these observations, showing highest chloroplast/cytoplasm ratios for the EPYC1 construct, with both EPYC1 and VIPP1 having significantly higher values than the linker and RBCXa constructs.

## Discussion

By definition, targeting peptides comprise the sequence that is both necessary for targeting a native cargo protein to its subcellular destination, and sufficient to redirect a heterologous cargo to the same location (Chotewutmontri et al., 2017). The present study demonstrates that in Chlamydomonas, the amino-terminal sequence required to specify chloroplast targeting significantly exceeds the cleavable part for eight out of ten tested cTPs. Based on the position of cleavage sites, algal cTPs have been noted in the past to be much shorter than their counterparts in vascular plants (Franzén et al., 1990). Yet the requirement for post-cleavage site residues implies that the amino-terminal sequences contributing to targeting in Chlamydomonas are in fact longer than recognized to date, with sequence elements that play a role in import present past the cleavage site just as in short plant cTPs (Caspari et al., 2021). With lengths of 56 and 60 residues, respectively, the import competent RBCA-cTP + 23 and RBCS-cTP + 23 constructs mirror the ~55–60 amino acid requirement of Arabidopsis cTPs (Bionda et al., 2010).

Previous studies in vascular plants suggested that the contribution of mature protein N-termini toward targeting may simply be a supply of unstructured sequence (Bionda et al., 2010; Shen et al., 2017). Bionda et al. (2010) found that using tightly folded titin as cargo protein prevented chloroplast import by short cTPs unless first denatured. Shen et al. (2017) found that ~20 residues of unfolded sequence downstream of the cTP cleavage site were required to target two *E. coli* proteins into rice chloroplasts. In principle, the chloroplast import machinery is capable of unfolding any protein for post-translational import, driven by an ATP-powered motor complex in the chloroplast stroma (Chotewutmontri et al., 2017). However, initiation of import requires the cTP to reach across both TOC and TIC passively before being able to contact stromal motor proteins. It is this initiation step that is most likely disrupted by the presence of structured elements too close to the N-terminus (Bionda et al., 2010). While less tightly folded than titin, the Venus fluorescent reporter used as cargo protein in the present study contains an α-helix directly at the N-terminus (Rekas et al., 2002), which may conceivably impair this passive import setup in the absence of an unfolded spacer sequence. Mitochondrial import lacks similar constraints, as mitochondrial targeting peptides are guided across the outer membrane translocase using interactions with increasing affinity (Schatz, 1997; Fukasawa et al., 2017), and then make use of the proton motive force across the inner membrane to power import even before contacting the matrix-localized PAM complex that uses ATP to energize further import (Martin et al., 1991; Wiedemann and Pfanner, 2017). Consistently, the two mTPs that were tried both achieved mitochondrial targeting without any sequence extension past the cleavage site.

The idea that unstructured sequence past the cleavage site may help short cTPs during the passive setup phase of import is supported by the finding that unstructured post-cleavage site sequence stretches were associated with shorter cTPs in Chlamydomonas. Longer cTPs would be expected to be less reliant on post-cleavage site residues, consistent with the finding that the comparatively long cTP of PSRP3 (84 residues) was able to support chloroplast import of Venus as a cTP + 5 construct. Studied cTPs were chosen to include both proteins related to photosynthesis and to plastid housekeeping, as import of these groups may be mediated by different receptors at the translocon (Chotewutmontri et al., 2017). The fact that members of both groups show import deficits in the cTP + 5 constructs (e.g., photosynthesis-related LHCB4, ATPC, and ribosomal protein PRPL4) points to the general import mechanism being affected, consistent with passive import setup being affected in the absence of a relatively unstructured sequence of sufficient length.

Perhaps most strikingly, chloroplast import by RCA1-cTP could be restored using unstructured sequences taken from chloroplast proteins EPYC1 and VIPP1 as post-cleavage site spacers. EPYC1 is an intrinsically disordered protein comprised of four near-identical internal repeats that act as linker protein within the chloroplast pyrenoid (Mackinder et al., 2016), a proteinaceous structure within the chloroplast that is central to the algal carbon concentrating mechanism (Caspari et al., 2017). VIPP1 plays a role in the biogenesis of thylakoid membranes and has an unstructured C-terminus (Zhang et al., 2016; Theis et al., 2019). C-terminal sequences from these proteins are not expected to contain any specific targeting motifs; thus, restoration of targeting lends further support to the importance of unstructured sequence at the N-terminus. In the same vain, copying an inherently structured sequence from the most C-terminal helix of RBCXa (Bracher et al., 2015) failed to restore efficient chloroplast import (Figure 3D).

An alternative explanation would require invoking the existence of specific sequence motifs that carry chloroplast targeting information beyond the cleavage site. Decades of research on cTPs have unearthed many such motifs prior to the cleavage site, but not much has been noted beyond, making this an unlikely proposition. In Chlamydomonas, Razzak et al. (2017) report the presence of such a motif (Q|MMVW) required for targeting in the Chlamydomonas RBCS-cTP in a location that spans the cleavage site (Schmidt et al., 1979). However, it is possible that affecting the cleavage site itself might either affect import itself or else prevent reporter accumulation in the chloroplast by targeting preproteins that retain a cTP for degradation. The fact that the efficiency of import improves gradually as mature protein residues are added to RCA1-cTP and RBCS2-cTP also appears more consistent with the idea of more unstructured sequence facilitating passive import setup to a greater extent; addition of specific targeting motifs might be expected to lead to step-changes in import efficacy.

Nonetheless, the PGP1-cTP + 5 construct failed to deliver Venus to the chloroplast, suggesting that downstream sequence plays a role even in a case like this where the mature protein N-terminus is not predicted to be unstructured. Conversely, the PSAD-cTP achieved targeting even in the absence of relatively unstructured downstream native sequence, even though this cTP should be too short (35 residues) to reach into the stroma passively. Presumably, the cargo protein is kept in an unfolded state prior to import, e.g., through recruitment of chaperones interacting with the cTP, or perhaps through co-translational import following polysome recruitment to the chloroplast envelope by this cTP. Finally, a G-rich linker failed to restore efficient chloroplast import, suggesting that this artificial flexible sequence differs in important ways from native unstructured sequences. These observations highlight the importance of specific sequence motifs and properties for import, and suggest it may be worth exploring the contribution of mature protein elements to targeting further. Indeed, Rolland et al. (2016) found that chloroplast import efficacy depended not only on the cTP, but also on the nature of the cargo protein. For example, while extended RBCS cTPs were able to target a GFP reporter to the chloroplast, the same sequences failed to generate import of more hydrophobic cargo proteins containing multiple transmembrane domains (Rolland et al., 2016).

An overall picture thus emerges that many of the short algal cTPs likely contain the information for chloroplast targeting upstream of the cleavage site but rely on additional unstructured sequence at mature protein N-termini to allow import to be successfully initiated. The importance of sufficient length and the presence of unstructured sequence elements for chloroplast targeting was equally evident in recent work aimed at generating *bona fide* cTPs from antimicrobial peptides (Caspari et al., 2021). Additional complexities exist and may warrant further research on the contribution of mature protein elements to chloroplast import, and how they can be circumvented by some targeting sequences such as in the case of PSAD. Indeed, for the purposes of sending heterologous proteins to the chloroplast (Crozet et al., 2018), the PSAD-cTP is likely the best choice among Chlamydomonas cTPs studied so far to ensure targeting through addition of a short sequence and without a need for additional constraints at the mature protein N-terminus.

## Data Availability Statement

The original contributions presented in the study are included in the article/supplementary material, further inquiries can be directed to the corresponding author.

## Author Contributions

The author confirms being the sole contributor of this work and has approved it for publication.

## Funding

Financial support from the Rothschild Foundation, LabEx Dynamo (ANR-LABX-011), and the ChloroMitoRAMP grant (ANR-19-CE13-0009) to OC, as well as annual funding from CNRS and Sorbonne University to UMR7141, is gratefully acknowledged.

## Conflict of Interest

The author declares that the research was conducted in the absence of any commercial or financial relationships that could be construed as a potential conflict of interest.

## Publisher’s Note

All claims expressed in this article are solely those of the authors and do not necessarily represent those of their affiliated organizations, or those of the publisher, the editors and the reviewers. Any product that may be evaluated in this article, or claim that may be made by its manufacturer, is not guaranteed or endorsed by the publisher.
